# Platelet-Lymphocyte and Neutrophil-Lymphocyte Ratio for Prediction of Hospital Outcomes in Patients with Abdominal Trauma

**DOI:** 10.1155/2022/5374419

**Published:** 2022-02-07

**Authors:** Ayman El-Menyar, Ahammed Mekkodathil, Amani Al-Ansari, Mohammad Asim, Eman Elmenyar, Sandro Rizoli, Hassan Al-Thani

**Affiliations:** ^1^Department of Surgery, Clinical Research, Trauma & Vascular Surgery, Hamad General Hospital, Doha, Qatar; ^2^Department of Clinical Medicine, Weill Cornell Medical College, Doha, Qatar; ^3^Department of Surgery, Trauma Surgery, Hamad General Hospital, Doha, Qatar; ^4^Bahcesehir University, Istanbul, Turkey; ^5^Department of Surgery, Trauma Surgery and Vascular Surgery, Hamad General Hospital, Doha, Qatar

## Abstract

**Background:**

The platelet-to-lymphocyte ratio (PLR) and neutrophil-to-lymphocyte ratio (NLR) reflect the patient inflammatory and immunity status. We investigated the role of on-admission PLR and NLR in predicting massive transfusion protocol (MTP) activation and mortality following abdominal trauma.

**Methods:**

A 4-year retrospective analysis of all adult abdominal trauma patients was conducted. Patients were classified into survivors and nonsurvivors and low vs. high PLR. The discriminatory power for PLR and NLR to predict MTP and mortality was determined. Multivariate logistic regression analysis was performed for predictors of mortality.

**Results:**

A total of 1199 abdominal trauma patients were included (18.7% of all the trauma admissions). Low PLR was associated with more severe injuries and greater rates of hospital complications including mortality in comparison to high PLR. On-admission PLR and NLR were higher in the survivors than in nonsurvivors (149.3 vs. 76.3 (*p* = 0.001) and 19.1 vs. 13.7 (*p* = 0.009), respectively). Only PLR significantly correlated with injury severity score, revised trauma score, TRISS, serum lactate, shock index, and FASILA score. Optimal cutoffs of PLR and NLR for predicting mortality were 98.5 and 18.5, respectively. The sensitivity and specificity of PLR were 81.3% and 61.1%, respectively, and 61.3% and 51.3%, respectively, for NLR. The AUROC for predicting MTP was 0.69 (95% CI: 0.655–0.743) for PLR and 0.55 (95% CI: 0.510–0.598) for NLR. To predict hospital mortality, the area under the curve (AUROC) for PLR was 0.77 (95% CI: 0.712–0.825) and 0.59 (95% CI: 0.529–0.650) for the NLR. On multivariate logistic regression analysis, the age, Glasgow Coma Scale, sepsis, injury severity score, and PLR were independent predictors of mortality.

**Conclusion:**

On-admission PLR but not NLR helps early risk stratification and timely management and predicts mortality in abdominal trauma patients. Further prospective studies are required.

## 1. Introduction

Traumatic injury is associated with significant morbidity and mortality and remains a public health problem worldwide [1]. The abdomen is the third most frequently injured body region, in which a quarter of patients may require explorative surgery [2]. Risk prediction of trauma is important for effective resource allocation, quality assessment, timely intervention, and estimation of patient outcomes [3]. In addition to the widely used risk scores that rely on the physiological and anatomical information such as the injury severity score (ISS), revised trauma score (RTS), and trauma and injury severity score (TRISS), several laboratory markers have been tested either individually or as a part of some scoring tools for predicting the patient outcomes [4–9]. These markers include hemoglobin, creatinine, base excess, activated partial thromboplastin time (PTT), prothrombin time (PT), international normalized ratio (INR), and serum lactate [8–11].

The role of platelets in the hemostasis and coagulation is crucial [12]. Platelets interaction with lymphocytes, neutrophils, and monocytes modifies both the innate and adaptive immune responses [13]. Platelets stick to the damaged endothelium and recruit leukocytes to the sites of injury [14]. Lymphocytes are the major cellular components of the humoral and cell-mediated immune system which include T, B, and natural killer cells [15]. Platelets, as a contributor to the inflammatory response, and platelet-associated chemokines such as platelet factor 4 and connective tissue-activating peptide III can modulate the inflammation process; however, low lymphocyte counts may lead to inadequate immune responses [16, 17].

The neutrophil and lymphocyte counts are expected to increase or decrease in the stressful and systemic inflammation conditions such as trauma and surgical interventions [17–20]. Furthermore, the platelet-to-lymphocyte ratio (PLR) could reflect the balance between the body response to inflammation and immunity mediators [16, 17].

The utility of PLR and neutrophil-to-lymphocyte ratio (NLR) as prognostic factors has been tested in several acute medical conditions such as pancreatitis, gastrointestinal bleeding, pulmonary embolism, sepsis, and acute cardiovascular diseases [21–25]. Moreover, a recent data showed that both NLR and PLR are reliable preoperative predictive markers of the postoperative overall survival and recurrence-free survival in patients with hepatocellular carcinoma [26]. Previously, few studies have tested the usefulness of PLR and NLR for predicting the outcomes in trauma patients [27–32]. However, the use of these ratios has not yet been evaluated in patients with abdominal trauma. Therefore, we aim to study the prognostic value of these biomarkers in abdominal trauma. The present study hypothesized that the on-admission PLR and NLR values would help early risk stratification and timely management of abdominal trauma patients which subsequently improve the outcomes.

## 2. Materials and Methods

A retrospective study was conducted among all adult patients who were admitted to the Hamad Trauma Center (HTC) between 2014 and 2017 due to abdominal trauma. The study was based on data obtained from the trauma registry at the HTC and the electronic medical records at the hospital. The HTC is the only level I trauma center in the country which sees and treats freely more than 95% of the trauma patients in the state of Qatar (the total country census is around 2.7 million). The trauma registry, which is the Qatar National Trauma Registry, contributes to and is compliant with the National Trauma Data Bank (NTDB) and Trauma Quality Improvement Program (TQIP) of the American College of Surgeons Committee on Trauma. Patients diagnosed with abdominal trauma (i.e., abdominal abbreviated injury score (AIS) > 1), and aged ≥18 years old at the time of admission were included in this study. Patients with non-abdominal trauma, pediatric group, patients with inaccessible or missing data from the hospital system or those who were brought in dead were excluded.

The collected data included patient demographics: mechanism of injury, Glasgow Coma Score (GCS) in the trauma room, abdominal injuries (liver, spleen, kidney, adrenal, pancreas, and pelvic hematoma), extra-abdominal injuries (head, chest, spine, arm, leg, pelvis, rib fracture, and femur fracture), revised trauma score (RTS), injury severity score (ISS), trauma injury severity score (TRISS), initiation of massive transfusion protocol (MTP), interventions, and length of stay (LOS) in the intensive care unit (ICU) and hospital. The laboratory findings include hemoglobin, white blood cells (WBC), platelet, lymphocytes, neutrophils, serum lactate, international normalized ratio (INR), platelet-to-lymphocyte ratio (PLR), and neutrophil-to-lymphocyte ratio (NLR). The PLR was calculated through the division of the absolute platelet count by the lymphocyte count, and NLR was calculated through the division of the absolute neutrophil count by the absolute lymphocyte count. We collected the first three blood test readings that were taken on arrival to the trauma room in the ED; however, we used the first reading for the study analysis. The FASILA score is the sum of the following parameters: Focused Assessment with Sonography in Trauma (FAST scan) result (negative = 0 and positive = 1), shock index (SI) value (0 = 0.50‐0.69, 1 = 0.70‐0.79, 2 = 0.80‐0.89, and 3 ≥ 0.90), and initial serum lactate reading (0 ≤ 2.0, 1 = 2.0‐4.0, and 2 ≥ 4.0 mmol/l). The minimum score is 0, and the maximum score is six; the value above 4 indicates the need of aggressive management and is associated with worse outcome [8]. The shock index is defined as the heart rate divided by the simultaneous systolic blood pressure on admission [9]. MTP is the prompt access to blood products in a balanced ratio. It was developed to optimize specific component replacement in a setting of severe hemorrhage (i.e., delivery of plasma-platelet-RBC in a ratio of 1 : 1 : 1) [33]. One MTP shipment was defined as the infusion of ≥6 units 5of packed RBC, ≥6 units of plasma, and ≥6 units of platelets [34].

### 2.1. Statistical Analysis

Data were presented as numbers, percentages, mean ± standard deviation, median, and range—whenever appropriate. The collected data were compared between survivor and nonsurvivor patient groups and between low and high PLR. Categorical variables were compared using the chi-square test. The Kolmogorov–Smirnov test was used to assess the normal distribution of the variables. Nonparametric parameters were analyzed using the Mann–Whitney *U* test. The means of continuous variables (normally distributed continuous variables) were compared using Student's *t*-test, whereas medians were compared using the nonparametric test. A receiver operating characteristic (ROC) curve was plotted to determine the discriminatory power of PLR and NLR to predict the MTP activation and in-hospital mortality. A cutoff value was defined as that with the highest validity. Sensitivity and specificity of PLR and NLR for the prediction of the MTP initiation and mortality in abdominal trauma patients were presented. The area under the curve (AUROC) was calculated and presented with 95% confidence interval (CI). Multivariate logistic regression analysis was performed to predict in-hospital mortality using the relevant and significant variables such as age, gender, GCS, ISS, shock index, sepsis, serum lactate, PLR, and NLR. Data were expressed as odds ratio (OR) and 95% confidence interval (CI). The 2-tailed *p* value less than 0.05 was considered statistically significant. The Statistical Package for the Social Sciences (SPSS) for Windows V.21.0 (SPSS, Chicago, Illinois, USA) was used for the data analysis. This study included the STROBE checklist (Supplementary Table (available here)).

### 2.2. Ethics Approval

This study was granted approval from the Research Ethics Committee of the Medical Research Center, Hamad Medical Corporation (IRB # MRC-01-18-003). A waiver of consent was granted as there was no direct contact with patients, and data were anonymously collected.

## 3. Results

A total of 1199 patients admitted at the HTC due to abdominal trauma were included in the study. This represents almost 18.7% of all the trauma admissions over 4 years. Of the total, 79 (6.6%) patients died in the hospital. The mean age of patients was 31 years, and over 90% were males. Most injuries were traffic-related (63%) followed by fall from height (20.4%). Blunt trauma was the main type of trauma (92.7%). Most of abdominal injuries involved the liver (35.8%) and spleen (27.6%). The main extra-abdominal injury was chest trauma (53.8%) (Table 1).

The mean ISS was 18. Intubation was performed in one out of the three patients. Blood transfusion was required in nearly 40%. Exploratory laparotomy was performed in 27% of patients, and open reduction internal fixation (ORIF) for associated bone fracture was needed in 17.8% patients. Pneumonia was reported as complication in 8.3%. The median LOS in the ICU was 5 days while that in the hospital was 7 days (Table 2).

Comparative analysis between survivors and nonsurvivors revealed that the traffic-related injuries led to more deaths (82.3% vs. 61.6%, *p* = 0.001). Nonsurvivors had significantly higher proportions of pancreatic injuries (13.9% vs. 1.8%, *p* = 0.001) while other abdominal injuries were comparable. Extra-abdominal injuries to the head, chest, spine, limb, and femur were higher among nonsurvivors than among survivors (*p* = 0.001) (Table 1). The ISS was higher in nonsurvivors (36.2 vs. 16.9, *p* = 0.001), whereas the RTS and TRISS were lower. All patients in the nonsurvivor group were intubated, whereas only 28.5% in the survivor group were intubated (*p* = 0.001). Blood transfusion was also higher in the nonsurvivors (*p* = 0.001). Splenectomy was more frequent in the nonsurvivors than in survivors (*p* = 0.004). In addition, complications such as pneumonia, sepsis, and acute respiratory distress syndrome (ARDS) were more common among the nonsurvivors (*p* = 0.001). The length of stay on the ventilator and ICU LOS were comparable between the study groups. Obviously, the hospital LOS was significantly longer in the survivors when compared to the nonsurvivors (8 vs. 5 days, *p* = 0.005) (Table 2).

Laboratory findings revealed that the mean PLR for survivors was significantly higher than that for the nonsurvivors (149.3 vs. 76.3, *p* = 0.001) (Table 3). Similarly, the mean NLR was significantly higher among survivors (19.1 vs. 13.7, *p* = 0.009). In the study cohort, MTP activation and exploratory laparotomy were required in 14% and 27% of cases, respectively. The PLR was significantly lower in patients who required MTP vs. no MTP (median72 (range 22–618) vs. 121 (range 14–940), *p* = 0.001). However, the difference was not statistically significant for those who required laparotomy vs. no laparotomy (106 (range 16–618) vs. 116 (range 14–940), *p* = 0.48).

### 3.1. Low vs. High PLR

Table 4 shows the comparison between low and high PLR based on the optimum cutoff by ROC (98.5). Low PLR was significantly associated with lower RTS and TRISS, higher ISS, and greater rates of positive FAST scan, exploratory laparotomy, MTP, splenectomy, hospital length of stay, and mortality (Table 4).

Table 5 shows the median (IQR) of PLR and NLR among the FASILA 7 scales. PLR was significantly decreasing along the increase in the FASILA scales (*p* = 0.002); it decreased from 142 and 128 with a scale of 0 and 1, respectively, to reach 80 and 74 in a scale of 5 and 6, respectively. NLR did not have statistically significant differences with the FASILA scales (*p* = 0.39).

The bivariate analysis showed significant correlations between PLR and variables such as GCS, blood transfused, ISS, RTS, the second reading of serum lactate, FASILA score, and TRISS (*p* < 0.05) (Table 6). On the other hand, NLR was not significantly correlated with any of these variables; however, PLR was significantly correlated with NLR (*r* = 0.63, *p* = 0.001).

On the multivariate analysis, age (OR 1.03; 95% CI 1.006-1.058, *p* = 0.01), GCS (OR 0.81; 95% CI 0.815-0.929, *p* = 0.001), sepsis (OR 4.4; 95% CI 1.63-12.18, *p* = 0.004), ISS (OR 1.09; 95% CI 1.06-1.12, *p* = 0.001), and on-admission PLR (OR 0.99; 95% CI 0.98-0.99, *p* = 0.03) were predictors of mortality whereas serum lactate, shock index, and NLR were not predictors for mortality (Table 7).

**Figure 1:**
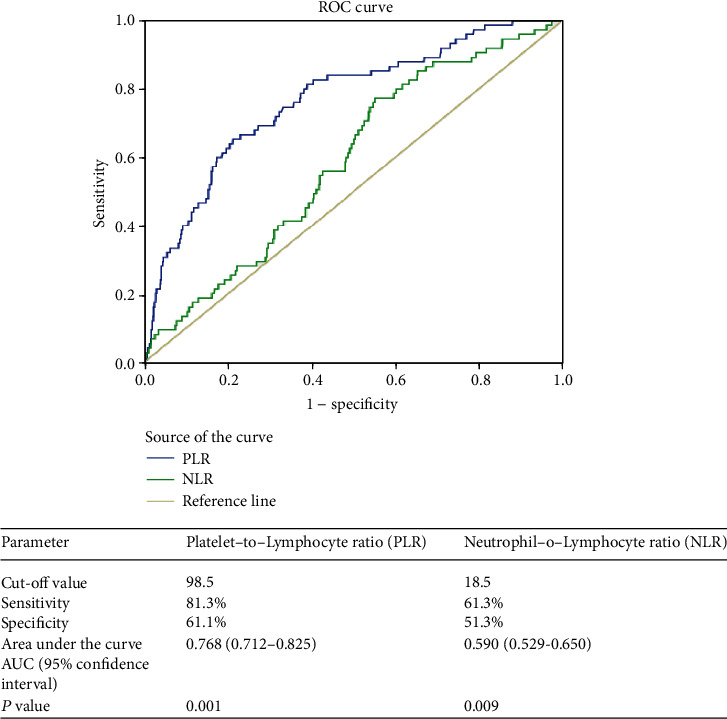
The receiver operating characteristic curve (ROC curve): cutoff value, sensitivity, and specificity of the platelet-to-lymphocyte ratio and neutrophil-to-lymphocyte ratio for prediction of mortality in abdominal trauma patients.

**Figure 2:**
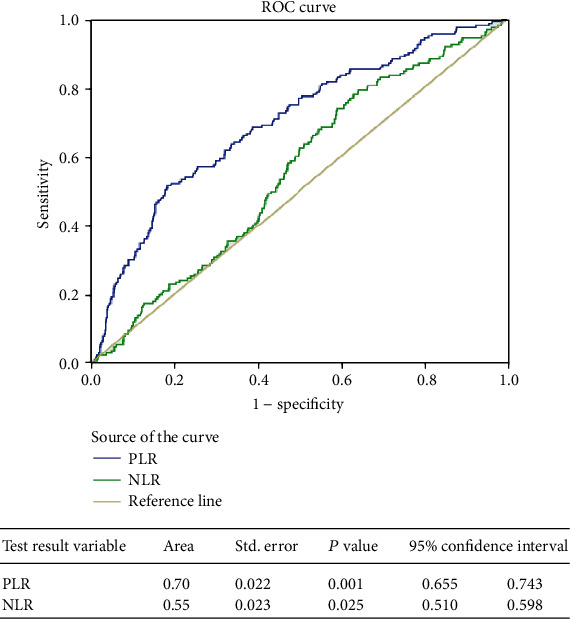
Area under the curve for predicting massive transfusion using PLR and NLR.

The ROC curve was plotted to assess the discriminative ability of PLR and NLR for mortality. For the PLR to predict mortality, the AUROC was 0.77 (95% CI (0.712-0.825)) while it was 0.59 (95% CI (0.529-0.650)) for the NLR. The optimal cutoff for predicting the hospital mortality for the PLR was 98.5, and that for the NLR was 18.5. Sensitivity and specificity for the PLR in predicting the mortality were 81.3% and 61.1%, respectively, while those for the NLR were 61.3% and 51.3%, respectively (Figure 1). The AUROC for predicting the MTP for PLR was 0.699 (95% CI (0.655-0.743)) (*p* = 0.001) (Figure 2). The AUROC for predicting the MTP for NLR was 0.554 (95% CI (0.510-0.598)) (*p* = 0.025).

## 4. Discussion

The PLR and NLR could reflect the inflammatory, immunity, and hemostatic status in acute illnesses [17–20]. To the best of our knowledge, this is the first study to evaluate the prognostic value of PLR and NLR in patients with abdominal trauma. The study demonstrates that PLR is a useful and simple bedside predictor of MTP activation and in-hospital mortality following abdominal trauma, while the NLR is a poor predictor. Low PLR is associated with more severe injuries and greater rates of hospital complications including mortality. The sensitivity and specificity of the PLR in predicting the mortality were 81.3% and 61.1%, respectively. Moreover, PLR did predict the MTP activation while NLR did not. The present study shows that there is a significant correlation between PLR and the injury severity scores (ISS, GCS, RTS, shock index, FASILA, and TRISS), transfused blood units, and the second reading of serum lactate. On the other hand, NLR does not show a significant correlation with these variables. Moreover, the multivariate analysis showed that PLR, but not the NLR, is age-, sex-, and GCS-adjusted predictor of the ISS and the development of sepsis and hospital mortality. However, PLR is more feasible and easier to get within the first hour in the ED. Studies focusing on NLR and PLR in predicting patient outcomes after abdominal trauma are lacking.  Table 8 summarizes the role of these ratios in other traumatic injury [27, 28, 30–32, 35–38].

Jo et al. [31] demonstrated that PLR was an independent predictive marker in adult patients with road traffic injuries. The study included 488 patients; mortality was nearly 9%. Abdominal trauma was reported in only 14% of the study cohort. The PLR values were lesser in the mortality group than in the survivor group (51.3 vs. 124.2, *p* < 0.001). The sensitivity and specificity for the PLR for the cutoff 85.6 were 90.7% and 35.5%, respectively.

Ke et al. [30] also reported that deceased adult trauma patients had significantly lower PLR values than the survivors (124.3 vs. 150.6, *p* = 0.001). The study included 2854 adult trauma patients in which the mortality was nearly 12% [30]. Moreover, there was no significant difference in NLR between the deceased and survivors. In our study, the PLR was significantly lower in the mortality group (76.3 vs. 149.3, *p* = 0.001), and the NLR was also significantly lower (13.7 vs. 19.1, *p* = 0.009). In contrast, Tekin [32] demonstrated that NLR (6.2 vs. 2.6, *p* < 0.001) and PLR (145.3 vs. 46.2, *p* < 0.001) were significantly higher in survivors than in the nonsurvivor group. The majority of patients had traffic-related injuries (82%). Seventy-eight (22%) patients underwent surgery in which abdominal surgery was one of the frequent surgeries. The cutoff value for NLR was 2.77 with sensitivity of 70% and specificity of 77%. For PLR, the cutoff was 61.83 with a sensitivity of 90% and specificity of 85%. The study demonstrated that NLR (OR, 3.2; *p* = 0.048) and PLR (OR, 0.90; *p* = 0.03) were independent predictors of mortality in pediatric trauma patients [32].

Dilektasli et al. [38] studied 1356 blunt trauma patients (≥16 years of age) admitted to the surgical intensive care unit to identify the predictive role of NLR. The investigators found that NLR greater than 8.19 and 7.92 was independently associated with in-hospital mortality at days 2 (hazard ratio 1.602, *p* = 0.019) and 5 (hazard ratio 3.758, *p* < 0.001), respectively.

Duchesne et al. [37] conducted a multi-institutional study on adult trauma patients who had severe hemorrhage and received MTP. The authors found that NLR was strongly associated with early mortality. Moreover, Cox regression models failed to show an NLR over 8.81 as a predictive of in-hospital mortality at day 3 (*p* = 0.056) but was predictive of mortality if NLR was greater than 13.68 at day 10 (*p* = 0.03).

Emektar et al. [27] investigated the effect of NLR and PLR on one-year mortality in old patients presented with hip fractures. The median NLR was 6.6 and 7.2 in the survivor and nonsurvivor groups, respectively (*p* = 0.04). On the other hand, the median PLR was 178 vs. 197 in the survivor and nonsurvivor groups, respectively (*p* = 0.02). The cutoff for NLR was 3.9 (sensitivity, 80%; specificity, 25%), and that for the PLR was 131 (sensitivity, 80%; specificity 30%). In this study, the platelet count was similar between the nonsurvivor and survivor groups, and the lymphocyte count was lower in the nonsurvivor group. In our study, the platelet count was significantly lower in the nonsurvivors and the lymphocyte count was higher.

Chae et al. [35] studied the prognostic value of the NLR and the neutrophil-to-lymphocyte platelet (NLPR) ratios among the adult trauma patients who underwent emergency surgery. This study concluded that NLPR, as a reflection of the systemic inflammatory condition, at day 7 was a superior predictor of late mortality than the other trauma scores.

Gameiro et al. [39] demonstrated that a higher postoperative NLPR was independently associated with acute kidney injury (AKI) after major abdominal surgery, while there was no association with in-hospital mortality. Ntalouka et al. [40] reported that the postoperative NLR and PLR were associated with the occurrence of AKI after endovascular aneurysm repair for abdominal aortic aneurysm. The postoperative NLR value of 9.9 was associated with the occurrence of AKI with a sensitivity of 80% and specificity of 81%, while the PLR value of 22.8 had sensitivity of 80% and specificity of 83%. Our prior work showed that in patients with repaired perforated peptic ulcer, the optimal cutoff value of PLR was 311.2 with AUC 0.702 and negative predictive value of 93% for the prediction of prolonged hospitalization (>1 week) [17].

Although the present study focused only on abdominal trauma and the sample size was high, its retrospective design was the main limitation. In addition, the study was single-centered, and therefore, the generalizability was under question. The lack of other inflammatory and immunity markers is also a limitation. One of the main utility of scoring systems is patient and family involvement and explanation about what to expect; however, the present retrospective analysis did not capture such information. Notably, most patients were males (90%) and the mechanism of injury was blunt trauma (93%), which reflects the pattern of trauma in Qatar [41]. Therefore, we cannot exploit the study findings in all genders and those who sustained penetrating injuries.

Lastly, the reported cutoffs in this study were different than those reported in other studies that were conducted on nontrauma patients such as oncology-based studies [42]. This observation may indicate the possible different roles of these indices (neutrophils, lymphocytes, NLR, and PLR) in trauma in comparison to the nontrauma patients and urges further prospective studies including other inflammatory markers as well (i.e., interleukin and vascular endothelial growth factor). These studies could explain whether the neutropenia, neutrophilia, lymphopenia, lymphocytosis, or thrombocytopenia is the underlying cause of having such ratios and impact.

## 5. Conclusions

On-admission PLR but not the NLR would help early risk stratification and timely management of abdominal trauma patients and subsequently reduce the in-hospital mortality. Further prospective studies are required to support the study findings.

## Figures and Tables

**Table 1:** Demographics and clinical presentations in survivors and nonsurvivors after abdominal trauma.

Variable	Overall (*n* = 1199)	Survivors (*n* = 1120)	Nonsurvivors (*n* = 79)	*p* value
Age	30.8 ± 13.5	30.7 ± 13.4	33.8 ± 14.9	0.07
Males	1081 (90.2%)	1010 (90.2%)	71 (89.9%)	0.93
Mechanism of injury				
Traffic related	755 (63.0%)	690 (61.6%)	65 (82.3%)	0.001 for all
Fall from height	245 (20.4%)	232 (20.7%)	13 (16.5%)	
Assault	66 (5.5%)	65 (5.8%)	1 (1.3%)	
Fall of heavy object	53 (4.4%)	53 (4.7%)	0 (0.0%)	
Others	80 (6.7%)	80 (7.1%)	0 (0.0%)	
Trauma type				
Blunt	1111 (92.7%)	1033 (92.2%)	78 (98.7%)	0.03 for all
Penetrating	88 (7.3%)	87 (7.8%)	1 (1.3%)	
On-admission vital signs				
TRU systolic blood pressure	118.3 ± 22.8	119.1 ± 21.9	107.2 ± 30.5	0.001
TRU diastolic blood pressure	74.3 ± 16.6	74.7 ± 16.1	69.0 ± 22.8	0.03
TRU pulse rate	97.4 ± 23.9	96.2 ± 23.2	114.6 ± 26.6	0.001
TRU respiratory rate	20.3 ± 5.2	20.3 ± 5.2	21.2 ± 5.4	0.18
TRU oxygen saturation	97.8 ± 5.9	98.2 ± 5.5	93.3 ± 9.8	0.001
TRU Glasgow Coma Scale	15 (3–15)	15 (3–15)	3 (3–15)	0.001
Extra-abdominal injuries				
Head	264 (22.0%)	201 (17.9%)	63 (79.7%)	0.001
Chest	645 (53.8%)	576 (51.4%)	69 (87.3%)	0.001
Pelvis	327 (27.3%)	301 (26.9%)	26 (32.9%)	0.24
Abdominal injuries				
Liver	429 (35.8%)	400 (35.7%)	29 (36.7%)	0.85
Spleen	331 (27.6%)	306 (27.3%)	25 (31.6%)	0.40
Kidney	184 (15.3%)	170 (15.2%)	14 (17.7%)	0.54
Adrenal	69 (5.8%)	63 (5.6%)	6 (7.6%)	0.46
Pancreas	31 (2.6%)	20 (1.8%)	11 (13.9%)	0.001

TRU: trauma room in the emergency department (ED).

**Table 2:** Injury severity, intervention, and complications.

Variable	Overall (*n* = 1199)	Survivors (*n* = 1120)	Nonsurvivors (*n* = 79)	*p* value
RTS	7.27 ± 1.26	7.41 ± 1.07	5.05 ± 1.82	0.001
TRISS	0.933 ± 0.164	0.951 ± 0.128	0.581 ± 0.311	0.001
ISS	18.2 ± 11.8	16.9 ± 10.9	36.2 ± 10.5	0.001
Intubation	398 (33.2%)	319 (28.5%)	79 (100%)	0.001
Positive FAST scan	345 (30.5%)	311 (29.5%)	34 (45.3%)	0.009
Exploratory laparotomy	326 (27.2%)	291 (26.0%)	35 (44.3%)	0.001
Massive transfusion protocol	170 (14.2%)	122 (10.9%)	48 (60.8%)	0.001
Blood transfusion	477 (39.8%)	402 (35.9%)	75 (94.9%)	0.001
ORIF for long bone fracture	213 (17.8%)	211 (18.8%)	2 (2.5%)	0.001
Splenectomy	66 (5.5%)	56 (5.0%)	10 (12.7%)	0.004
Complications				
Abdominal compartment syndrome	4 (0.3%)	1 (0.1%)	3 (3.8%)	0.001
Disseminated intravascular coagulopathy	4 (0.3%)	3 (0.3%)	1 (1.3%)	0.13
Pneumonia	100 (8.3%)	82 (7.3%)	18 (22.8%)	0.001
Sepsis	38 (3.2%)	28 (2.5%)	10 (12.7%)	0.001
Acute respiratory distress syndrome	30 (2.5%)	18 (1.5%)	12 (15.2%)	0.001
Total hospital LOS	7 (1-505)	8 (1-505)	5 (1-138)	0.005
Intensive care LOS	5 (1-161)	5 (1-161)	8 (1-81)	0.08
Ventilatory days	5 (1-73)	6 (1-49)	5 (1-73)	0.82

RTS: revised trauma score; TRISS: trauma injury severity score; ISS: injury severity score; LOS: length of stay; FAST: Focused Assessment with Sonography in Trauma; ORIF: open reduction and internal fixation.

**Table 3:** Laboratory findings in the study groups.

Initial laboratory findings	Overall (*n* = 1199)	Survivors (*n* = 1120)	Nonsurvivors ($\underset{¯}{n}=79$)	*p* value
Hemoglobin	13.3 ± 4.1	13.4 ± 4.2	10.9 ± 2.3	0.001
White blood count	15.7 ± 6.6	15.7 ± 6.6	16.1 ± 6.9	0.59
Platelet count	258.3 ± 86.6	261.6 ± 86.5	209.2 ± 72.6	0.001
Lymphocyte count	2.6 ± 1.7	2.5 ± 1.7	3.6 ± 1.9	0.001
Neutrophil count	49.3 ± 32.5	49.2 ± 32.6	49.6 ± 30.4	0.91
Serum lactate	2.7 (0.4‐220)	2.6 (0.4‐220)	4.3 (1.2‐14.9)	0.001
International normalized ratio (INR)	1.2 ± 0.5	1.1 ± 0.3	1.8 ± 1.3	0.001
Platelet-to-lymphocyte ratio	144.7 ± 133.0	149.3 ± 135.7	76.3 ± 48.1	0.001
Neutrophil-to-lymphocyte ratio	18.7 (0.4‐467.5)	19.1 (0.4‐467.5)	13.7 (0.5‐128.7)	0.009

**Table 4:** Injury severity and complications based on the platelet-lymphocyte ratio.

	Low PLR < 98.5 (*n* = 489, 41.5%)	High PLR ≥ 98.5 (*n* = 689, 58.5%)	*p* value
Age	30.2 ± 13.4	31.2 ± 13.6	0.26
Revised trauma score (RTS)	6.9 ± 1.5	7.5 ± 0.9	0.001
Trauma and injury severity score (TRISS)	0.89 ± 0.2	0.95 ± 0.12	0.001
Injury severity score (ISS)	21.3 ± 13.1	16.0 ± 10.4	0.001
FAST positive	150 (32.6%)	190 (29.2%)	0.29
Exploratory laparotomy	151 (30.9%)	171 (24.8%)	0.02
Massive blood transfusion	111 (22.7%)	55 (8.0%)	0.001
Blood transfusion	253 (51.7%)	216 (31.3%)	0.001
Transfused blood units	6 (1-79)	4 (1-73)	0.03
Intubation	226 (46.2%)	165 (23.9%)	0.001
ORIF surgery	93 (19.0%)	118 (17.1%)	0.40
Splenectomy	36 (7.4%)	30 (4.4%)	0.02
Complications			
Pneumonia	54 (11.0%)	43 (6.2%)	0.003
Sepsis	15 (3.1%)	22 (3.2%)	0.90
Acute respiratory distress syndrome	16 (3.3%)	12 (1.7%)	0.08
Total hospital length of stay	24 (1-193)	23 (1-158)	0.001
ICU length of stay	10 (1-161)	10 (1-81)	0.002
Ventilatory days	6 (1-41)	6 (1-73)	0.71
Mortality	61 (12.5%)	14 (2.0%)	0.001

**Table 5:** PLR and NLR among FASILA scores.

	FASILA scale							*p* value
	0	1	2	3	4	5	6	
PLR	142 (95-230)	128 (89-193)	108 (75-174)	123 (83-174)	101 (75-140)	80 (55-139)	74 (60-139)	0.002
NLR	21 (5-42)	20 (6-43)	17 (5-40)	23 (8-44)	15 (5-33)	14 (5-28)	18 (5-32)	0.386

Data presented as median and interquartile range. PLR: platelet-lymphocyte ratio; NLR: neutrophil-lymphocyte ratio.

**Table 6:** Bivariate correlations between biomarker ratios, injury severity scores, and transfused blood units.

	GCS	SI	Blood units transfused	ISS	RTS	TRISS	PLR	FASILA score	Lactate (1)	Lactate (2)
PLR										
Pearson's correlation	0.12^∗∗^	−0.10	−0.12^∗^	−0.11^∗∗^	0.11^∗∗^	0.07^∗^	1	−0.14	−0.03	−0.15
Sig. (2-tailed)	0.001	0.004	0.010	0.001	0.001	0.033	—	0.001	0.25	0.001
*N*	1145	1132	469	1168	1050	1025	1178	889	1062	590
NLR										
Pearson's correlation	0.011	−0.02	−0.084	−0.017	−0.019	−0.033	0.631^∗∗^	−0.03	− 0.005	−0.04
Sig. (2-tailed)	0.714	0.41	0.069	0.560	0.537	0.293	0.001	0.43	0.87	0.36
*N*	1143	1130	469	1166	1049	1024	1176	889	1061	590

Correlation is significant at the 0.01 level (2-tailed); lactate: (1) first reading of serum lactate and (2) second reading. RTS: revised trauma score; TRISS: trauma injury severity score; ISS: injury severity score; GCS: Glasgow Coma Scale; SI: shock index.

**Table 7:** Multivariate regression analysis for the predictors of mortality.

Variable	Odds ratio	95% Confidence interval		*p* value
Age in years^∗^	1.032	1.006	1.058	0.014
Sex (male)	0.573	0.177	1.856	0.353
On-admission GCS^∗^	0.870	0.815	0.929	0.001
Sepsis	4.452	1.627	12.179	0.004
Injury severity score (ISS)^∗^	1.087	1.056	1.118	0.001
Shock index^∗^	1.307	0.702	2.432	0.398
On-admission serum lactate^∗^	1.015	0.991	1.039	0.214
On-admission NLR^∗^	0.998	0.982	1.014	0.797
On-admission PLR^∗^	0.991	0.983	0.999	0.026

^∗^Continuous variable. GCS: Glasgow Coma Scale.

**Table 8:** Summary of studies on the NLR and PLR in trauma patients.

Author (year)	Study design/sample size	Trauma population	Predictors	Result
Wang et al. (2021) [28]; China	Retrospective (*n* = 460)	Elderly hip fracture patients (>60 y)	PLR	PLR was significantly higher in nonsurvivors than in survivors (*p* < 0.05), PLR was an independent predictor for one-year all-cause mortality (HR 1.56, 95% CI 1.02-2.41, *p* = 0.041)
Ke et al. (2021) [30]; Taiwan	Retrospective (*n* = 2854)	Adult trauma patients admitted to the ICU	MLR, NLR, PLR	Lower PLR for survivors than for nonsurvivors (124.3 vs. 150.6, *p* < 0.001), NLR was comparable
Chae et al. (2021) [35], Korea	Retrospective (*n* = 209)	>19 y who underwent emergency surgery after trauma	NLR, NLPR	NLPR at day 7 may be a superior predictor of late mortality compared with preexisting trauma scores
Jo et al. (2020) [31]; Korea	Retrospective (*n* = 488)	Adult traffic-related trauma admissions	PLR	Lower PLR in nonsurvivors than in survivors (51.3 vs. 124.2, *p* < 0.001)
Tekin (2019) [32]; Turkey	Retrospective (*n* = 358)	Pediatric trauma admissions	NLR, PLR	NLR and PLR were significantly higher in survivors than in nonsurvivors (NLR, 6.2 ± 5.7 versus 2.6 ± 2.5, *p* < 0.001; PLR, 145.3 ± 85.0 versus 46.2 ± 25.2, *p* < 0.001)
Chen et al. (2019) [36]; China	Retrospective (*n* = 316)	Traumatic brain injury	NLR	The day 1 NLR and admission GCS were independently correlated with increased peak NLR. Peak NLR was a predictor for 1-year outcomes
Duchesne et al. (2017); USA [37]	Retrospective (*n* = 285)	Severe hemorrhage patients received MTP	NLR	NLR > 13.68 at day 10 predicts mortality (*p* = 0.036)
Emektar et al. (2017) [27]; Turkey	Retrospective (*n* = 560)	Elderly hip fracture patients (>60 y)	NLR, PLR	For predicting 1 y mortality, the HR of NLR and PLR were 1.059 (1.022–1.097, *p* = 0.002) and 0.997 (0.994–0999, *p* = 0.01), respectively
Dilektasli et al. (2016) [38]; USA	Retrospective (*n* = 1356)	Trauma patients (>16 y) admitted to the ICU	NLR	NLR > 7.92 independently associated with in-hospital mortality at day 5 (HR 3.758, *p* < 0.001)
El-Menyar et al. (Qatar)^⁎^	Retrospective (*n* = 1199)	Adult patients with abdominal trauma	NLR, PLR	Initial PLR but not the NLR values after arrival at the trauma center would help early risk stratification and timely management of abdominal trauma patients

MLR: monocyte-to-lymphocyte ratio; NLR: neutrophil-to-lymphocyte ratio; PLR: platelet-to-lymphocyte ratio; NLPR: neutrophil-to-lymphocyte platelet ratio (NLPR). ^∗^The present study.

## Data Availability

All data have been given in the results and tables of the manuscript. Deidentified data can be requested from the Medical Research Center, Doha, Qatar, after signing a data sharing agreement.

## Supplementary Materials

Supplementary Materials Suppl table 1: STROBE Statement—checklist of items that should be included in reports of observational studies.
